# Determinants of the Mechanical Behavior of Human Lumbar Vertebrae After Simulated Mild Fracture

**DOI:** 10.1002/jbmr.264

**Published:** 2010-10-06

**Authors:** Julien Wegrzyn, Jean-Paul Roux, Monique E Arlot, Stéphanie Boutroy, Nicolas Vilayphiou, Olivier Guyen, Pierre D Delmas, Roland Chapurlat, Mary L Bouxsein

**Affiliations:** 1 INSERM Research Unit 831, Université de LyonLyon, France; 2 Department of Orthopaedic Surgery, Hôpital Edouard HerriotLyon, France; 3 Center for Advanced Orthopaedic Studies, Beth Israel Deaconess Medical Center and Harvard Medical SchoolBoston, MA, USA

**Keywords:** OSTEOPOROSIS, VERTEBRAL FRACTURE, VERTEBRAL STRENGTH, BIOMECHANICS, MICROARCHITECTURE

## Abstract

The ability of a vertebra to carry load after an initial deformation and the determinants of this postfracture load-bearing capacity are critical but poorly understood. This study aimed to determine the mechanical behavior of vertebrae after simulated mild fracture and to identify the determinants of this postfracture behavior. Twenty-one human L_3_ vertebrae were analyzed for bone mineral density (BMD) by dual-energy X-ray absorptiometry (DXA) and for microarchitecture by micro–computed tomography (µCT). Mechanical testing was performed in two phases: initial compression of vertebra to 25% deformity, followed, after 30 minutes of relaxation, by a similar test to failure to determine postfracture behavior. We assessed (1) initial and postfracture mechanical parameters, (2) changes in mechanical parameters, (3) postfracture elastic behavior by recovery of vertebral height after relaxation, and (4) postfracture plastic behavior by residual strength and stiffness. Postfracture failure load and stiffness were 11% ± 19% and 53% ± 18% lower than initial values (*p* = .021 and *p* < .0001, respectively), with 29% to 69% of the variation in the postfracture mechanical behavior explained by the initial values. Both initial and postfracture mechanical behaviors were significantly correlated with bone mass and microarchitecture. Vertebral deformation recovery averaged 31% ± 7% and was associated with trabecular and cortical thickness (*r* = 0.47 and *r* *=* 0.64; *p* = .03 and *p* *=* .002, respectively). Residual strength and stiffness were independent of bone mass and initial mechanical behavior but were related to trabecular and cortical microarchitecture (|*r*| = 0.50 to 0.58; *p* = .02 to .006). In summary, we found marked variation in the postfracture load-bearing capacity following simulated mild vertebral fractures. Bone microarchitecture, but not bone mass, was associated with postfracture mechanical behavior of vertebrae. © 2011 American Society for Bone and Mineral Research.

## Introduction

Vertebral fracture is the most common osteoporotic fracture, with one in three women and one in five men over the age of 50 years predicted to suffer a vertebral fracture in their remaining lifetime.(1) Moreover, after sustaining a vertebral fracture, the risk of all types of fractures increases significantly, and in particular, 20% of women will experience another vertebral fracture within the first year after their initial vertebral fracture.(2) Women with at least one mild vertebral fracture have a fourfold greater risk of subsequent vertebral fractures than those without prior fractures, and this risk increases dramatically with the number and severity of prior vertebral fractures.(3–5) This scenario of one vertebral fracture leading to another has been termed the *vertebral fracture cascade.*(6) Several factors may contribute to the vertebral fracture cascade, including altered spine kinematics owing to kyphosis, altered load transfer between adjacent vertebrae, and reduced activity after the initial fracture leading to disuse osteoporosis with accelerated bone loss.(7) However, despite many clinical and epidemiologic investigations, the mechanisms underlying progression of an existing vertebral deformity from mild to moderate or severe, as well as the factors contributing to vertebral fracture cascade, are poorly understood.(3,6,8–10) In particular, there is only limited information about the mechanical behavior of a vertebral body after an initial deformity or fracture, and it is of interest to understand the mechanical behavior of a fractured vertebra to better understand the vertebral fracture cascade.(6,9,10)

Thus the aims of this study were to determine the mechanical behavior of a human lumbar vertebra after initial mild fracture and to identify the factors that are associated with this postfracture mechanical behavior.

## Materials and Methods

### Bone specimens and bone mass assessment

Lumbar vertebrae (L_3_) were harvested fresh from 21 whole lumbar spines (L_1_ to L_5_) of human donors, including 11 men and 10 women aged 54 to 93 years (75 ± 10 years for men and 76 ± 10 years for women). Source of the donors was anatomic donation, and their available medical history was limited to the cause of the death. Specimens were obtained fresh and maintained frozen at −20°C wrapped in saline-soaked gauze until mechanical testing.(11,12)

The absence of prevalent fractures or significant bone diseases (ie, bone metastasis, Paget disease, or major osteoarthritis) involving the whole lumbar spine was confirmed by high-resolution lateral radiographs (Faxitron X-Ray Corporation, Lincolnshire, IL, USA) prior to L_3_ dissection. We evaluated lumbar osteoarthritis (OA) on the lateral radiographs according to the Kellgren-Lawrence (K/L) grading scale.(13) Severity of OA was assessed according to the presence of osteophytes and disk narrowing using a four-point scale: normal, minimal, moderate, or severe. Vertebrae with severe OA (grade 4) were excluded. Of those included in the study, 11 (52%), 8 (38%), and 2 (10%) were graded normal, minimal, or moderate OA, respectively.

Bone mineral content (BMC, g) and areal bone mineral density (aBMD, g/cm^2^) of the vertebral body were measured using dual-energy X-ray absorptiometry (DXA; Delphi W, Hologic, Waltham, MA, USA).

### µCT image acquisition and microarchitecture assessment

Image acquisitions of the whole vertebral body were performed using (1) a micro–computed tomography device (Skyscan 1076, Aartselaar, Belgium) with a nominal isotropic voxel size of 35 µm (field of view 70 mm, 2000 × 2000 pixels, X-ray source 100 kV, 100 µA) and (2) a high-resolution peripheral quantitative computed tomography device (HR-pQCT, XtremeCT, Scanco Medical AG, Bassersdorf, Switzerland) with a nominal isotropic voxel size of 82 µm (1536 × 1536 pixels, X-ray source 60 kV, 900 µA).

3D trabecular microarchitecture parameters were measured using direct methods (ie, distance-transformation algorithms that do not rely on assumptions about the underlying structure) and were designated with an asterisk.(14) The trabecular region of interest was defined manually in order to exclude cortical component of the vertebral body, as described in our previous studies.(14,15) The following trabecular microarchitecture parameters were measured: bone volume fraction (BV/TV, %), direct trabecular thickness (Tb.Th*, µm), degree of anisotropy (DA, 0 = isotropic; 1 = anisotropic), and structure model index (SMI, 0 = platelike; 3 = rodlike). The following cortical parameters were assessed: cortical thickness (Ct.Th, µm), cortical porosity (Ct.Po, %) defined by the canal area/cortical area ratio, and radius of curvature (Ct.Curv, mm) expressed by the mean of three 2D slice-scans using Morpho Expert Explora Nova software (La Rochelle, France).(15) All the previous parameters were measured using Skyscan data. However, since assessment of direct trabecular number (Tb.N*, *n*/mm), separation (Tb.Sp*, µm), and trabecular microarchitecture heterogeneity [ie, the standard deviation of Tb.Sp* on the entire vertebral trabecular volume (Tb.Sp*SD)] were not available with Skyscan Ant 3D analyzing software, these parameters were measured directly using Xtreme CT with the software developed for ex vivo analysis (Scanco Medical AG).(14)

### Mechanical testing

After thawing at room temperature (+20°C), soft tissues and posterior vertebral arches were removed. Then the midvertebral endplate-to-endplate height was measured using a caliper. Vertebral bodies were maintained at +4°C moist with Ashman's solution until mechanical testing.(11,12)

**Fig. 1:**
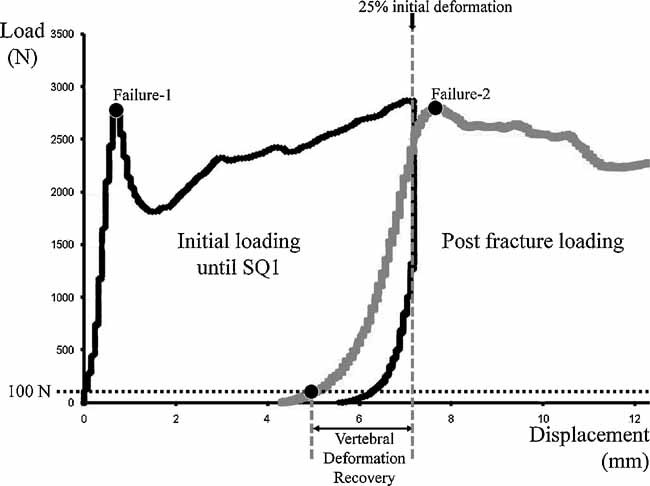
Load-displacement curves of an L3 vertebra. The black curve corresponds to the initial mechanical test performed with an initial loading until SQ1 fracture (25% deformation). The gray curve corresponds to the postfracture mechanical test performed after a 30-minute period of relaxation. Vertebral deformation recovery (VDR, %) corresponds to the height recovery with regard to the initial height. Failure 1 corresponds to the initial failure load and failure 2 to the postfracture failure load.

Mechanical testing was performed in two phases: The initial phase compressed the vertebra to create a mild vertebral fracture (25% deformation),(16) and the second phase, performed after a 30-minute unloaded period of relaxation, assessed the behavior of a vertebra after sustaining an initial mild fracture(9,10) (Fig. 1).

Before testing, a polyester resin interface (Soloplast V11, Vosschemie, Saint Egrève, France) with a quick-setting polymerization at low temperature (exothermic peak of resin polymerization ≤ +40°C) was applied to each endplate of the vertebral body to achieve parallel surfaces for load application. Preconditioning was performed prior to testing (10 cycles with loading at 100 N and unloading at 50 N). Then quasi-static uniaxial compressive testing was performed on the whole vertebral body submerged in Ashman's solution at controlled +37°C with a screw-driven materials-testing system (Schenck RSA-250, Darmstadt, Germany). Vertebrae were compressed to a height reduction of 25% using a constant displacement of 0.5 mm/min. This 25% deformation was chosen because it corresponds to grade I of semiquantitative (SQ1) assessment of vertebral fractures described by Genant and colleagues(16) and represents the most common osteoporotic vertebral fracture.(8,16,17) After 30 minutes of unloaded relaxation, a second uniaxial compressive test at the same constant displacement rate was performed—similar to the initial test—until a new 25% deformation was achieved (Fig. 1). The compressive load and displacement were assessed, respectively, by a 5000-N load cell (TME, F 501 TC) and a displacement transducer mounted directly on the vertebral resin endplates (Mécanium, Lyon, France).

We determined the following parameters from the two sets of load-displacement data: (1) initial and postfracture failure loads (N), defined as the peak force on the load-displacement curve, (2) initial and postfracture compressive stiffnesses (N/mm), defined by the linear part of the load-displacement curve slope between 25% to 75% of the failure load, and (3) initial and postfracture works to failure (N· mm), defined by the area under the load-displacement curve until failure load(18) (Fig. 1).

We calculated (1) changes in mechanical parameters (Δ, %), defined as the difference between postfracture and initial parameters and expressed as a percentage of the initial value, and (2) vertebral deformation recovery (VDR, %)—to explore postfracture elastic property of the vertebra. VDR is defined as the height recovery after relaxation relative to the initial height [VDR = (*H*_*r*_/*H*_25%_) × 100, with *H*_*r*_ (mm) = vertebral height recovery after relaxation and *H*_25%_ (mm) = vertebral height at mild fracture (ie, 25% deformation)]. VDR = 100% corresponds to a total height recovery after relaxation. We also calculated (3) residual strength and residual stiffness—to explore the postfracture plastic properties of the vertebra. Residual strength and stiffness were defined as the remaining load-bearing capacity after an initial fracture: Residual strength or stiffness = (postfracture parameter/initial parameter) × 100. Residual strength or stiffness = 100% corresponds to the absence of loss in load-bearing capacity (Fig. 1).

### Statistical analysis

Data are presented as the mean, SD, and range. The following tests were used: (1) Mann-Whitney tests for the comparison of variables between two groups, (2) Wilcoxon signed-rank tests for comparison between initial and postfracture mechanical variables, and (3) Spearman coefficients of correlation for analysis of the relationship between two variables. Results were considered significant if the *p* value was less than .05. All statistical analyses were performed using SPSS 16.0 (SPSS, Inc., Chicago, IL, USA).

## Results

Descriptive statistics for DXA and µCT parameters are shown in Table 1 and those for mechanical parameters in Table 2. There was no influence of age on microarchitectural and mechanical parameters except a trend for primary failure load to be negatively associated with age (*r* = −0.40, *p* = .07). The Kellgren-Lawrence (K/L) OA score did not differ between male and female donors, and there were no significant associations between K/L grades and BMD, microarchitecture, or mechanical parameters. Variables were similar in men and women, except that men had higher bone mineral content (BMC) than women (7.72 ± 1.96 g versus 5.76 ± 1.25 g, *p* = .014). The vertebral body height averaged 30 ± 3 mm (range 26.4 to 37.5 mm). There was no influence of vertebral height on initial and postfracture mechanical behaviors.

**Table 1:** Descriptive Statistics for DXA and Microarchitectural Parameters

	Mean ± SD	Range
DXA measurements		
BMC (g)	6.8 ± 1.91	2.96–9.68
BMD (g/cm^2^)	0.62 ± 0.12	0.36–0.80
µCT measurements		
BV/TV (%)	16 ± 4.43	8.78–25.85
DA (*n*)	0.43 ± 0.03	0.36–0.47
SMI (*n*)	1.79 ± 0.23	1.26–2.15
Tb.Th* (µm)	241 ± 42	188–329
Ct.Th (µm)	732 ± 445	319–1983
Ct.Po (%)	3.01 ± 3.26	0.20–12.43
Ct.Curv (mm)	33 ± 15	12–70
Tb.N* (*n*/mm)	0.76 ± 0.16	0.46–1
Tb.Sp* (µm)	1363 ± 332	972–2181
Tb.Sp*SD (*n*)	0.53 ± 0.16	0.31–1

**Table 2:** Descriptive Statistics for Initial and Postfracture Mechanical Parameters and Changes in Mechanical Parameters

	Failure load (N)		Stiffness (N/mm)		Work to failure (N.mm)	
Initial	2615 ± 1136	(651–5481)	2938 ± 1585	(663–6741)	1730 ± 1129	(453–4158)
Postfracture	2285* ± 970	(566–4547)	1277* ± 596	(156–2357)	3219* ± 1745	(654–7524)
Δ (%)	−11 ± 19	(−53–21)	−53 ± 18	(−76 to −2)	121 ± 104	(−34–425)

*Note:* Δ = difference between postfracture and initial parameters in % [mean ± SD (range)]. The comparisons between initial and postfracture mechanical parameters were performed using Wilcoxon signed-rank tests (*p < .05).

**Fig. 2:**
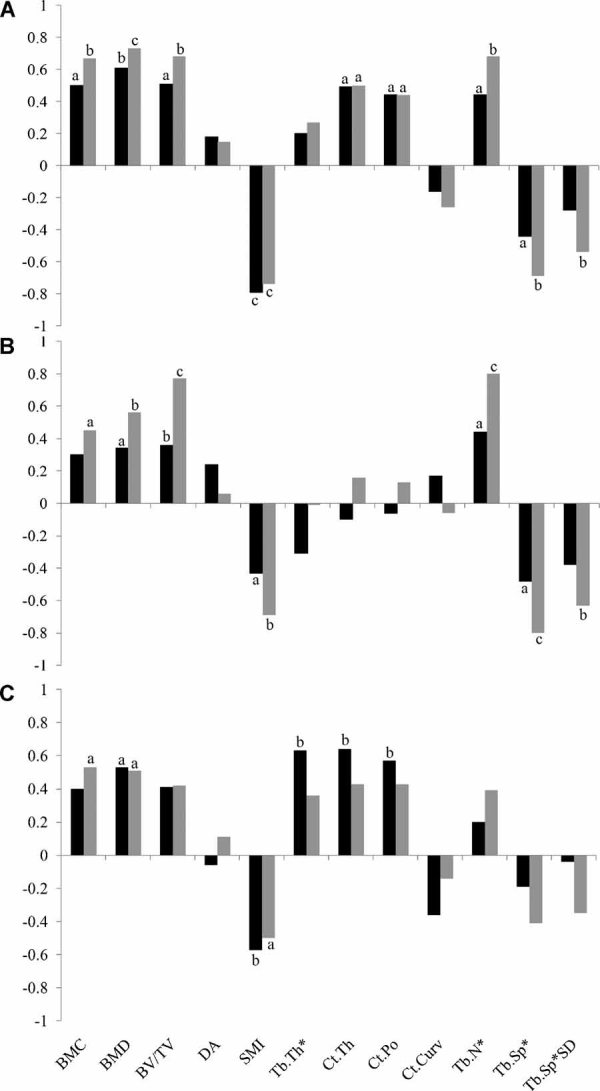
Spearman coefficients of correlation between initial mechanical parameters (black blocks), postfracture mechanical parameters (gray blocks), and microarchitecture. (A) Failure load, (B) stiffness, and (C) work to failure. a: *p* < .05; b: *p* < .01; c: *p* < .0001.

Initial mechanical behavior, as well as postfracture mechanical behavior, was correlated with bone mass and microarchitecture (Fig. 2).

### Relation between initial and postfracture mechanical behaviors

Postfracture failure load and postfracture stiffness were, respectively, 11% ± 19% and 53% ± 18% lower than initial values (*p* = .021 and *p* < .0001, respectively; Table 2). Postfracture work to failure was, on average, 121% ± 104% higher than initial value (*p* < .0001; Table 2).

Postfracture mechanical properties were significantly correlated with their corresponding initial values (*r* = 0.54 to 0.83, *p* < .0001 for failure load and stiffness and *p* = .012 for work to failure), with 29% of the variation in work to failure, 53% of the variation in stiffness, and 69% of the variation in failure load explained by the initial values.

### Mechanical properties of post-fracture vertebrae

#### Postfracture elastic property: vertebral deformation recovery (VDR, %)

**Fig. 3:**
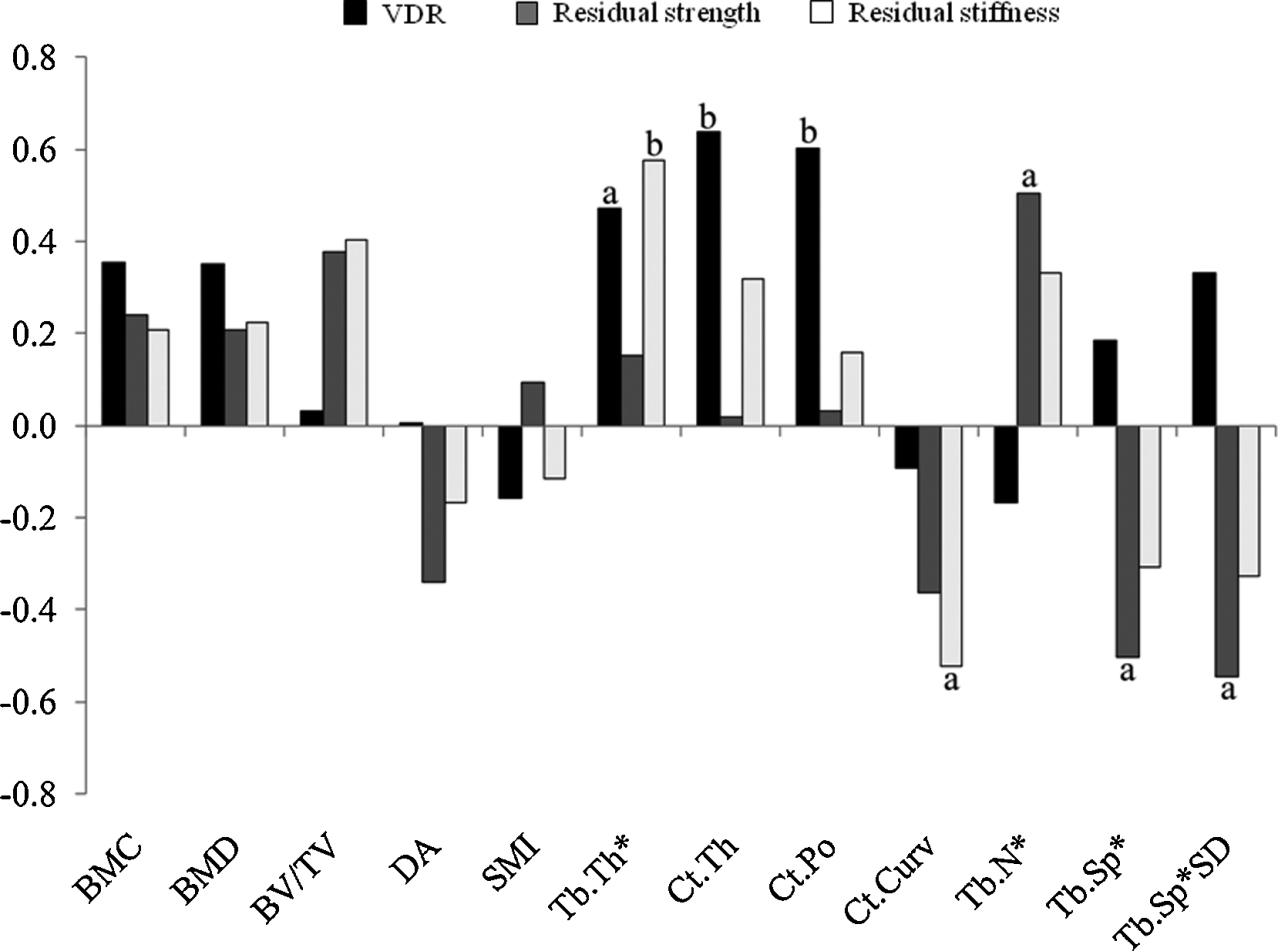
Spearman coefficients of correlation between residual mechanics and microarchitecture. (Black block) Vertebral deformation recovery (VDR); (gray block) residual strength, (white block) residual stiffness. a: *p* < .05; b: *p* < .01.

Vertebral deformation recovery averaged 31% ± 7% (range 20% to 46%) and was significantly and positively correlated with initial work to failure (*r* = 0.52, *p* = .016) but independent of bone mass parameters (ie, BMC, BMD, and BV/TV). In addition, VDR was significantly and positively correlated with Tb.Th* (*r* = 0.47, *p* = .03), Ct.Th (*r* = 0.64, *p* = .002), and Ct.Po (*r* = 0.60, *p* = .004; Fig. 3). Ct.Po was significantly and positively correlated with Ct.Th (*r* = 0.91, *p* < .0001).

#### Postfracture plastic property: residual strength and residual stiffness

Residual strength averaged 89% ± 19% (range 47% to 121%) of initial values. Residual strength was not correlated with bone mass (ie, BMD, BMC, and BV/TV) or with initial mechanical behavior. In addition, residual strength was significantly and positively correlated with Tb.N* (*r* = 0.50, *p* = .02) and significantly and negatively correlated with Tb.Sp* and Tb.Sp*SD (*r* = −0.50 and −0.55, *p* = .02 and *p* = .011, respectively; Fig. 3).

**Fig. 4:**
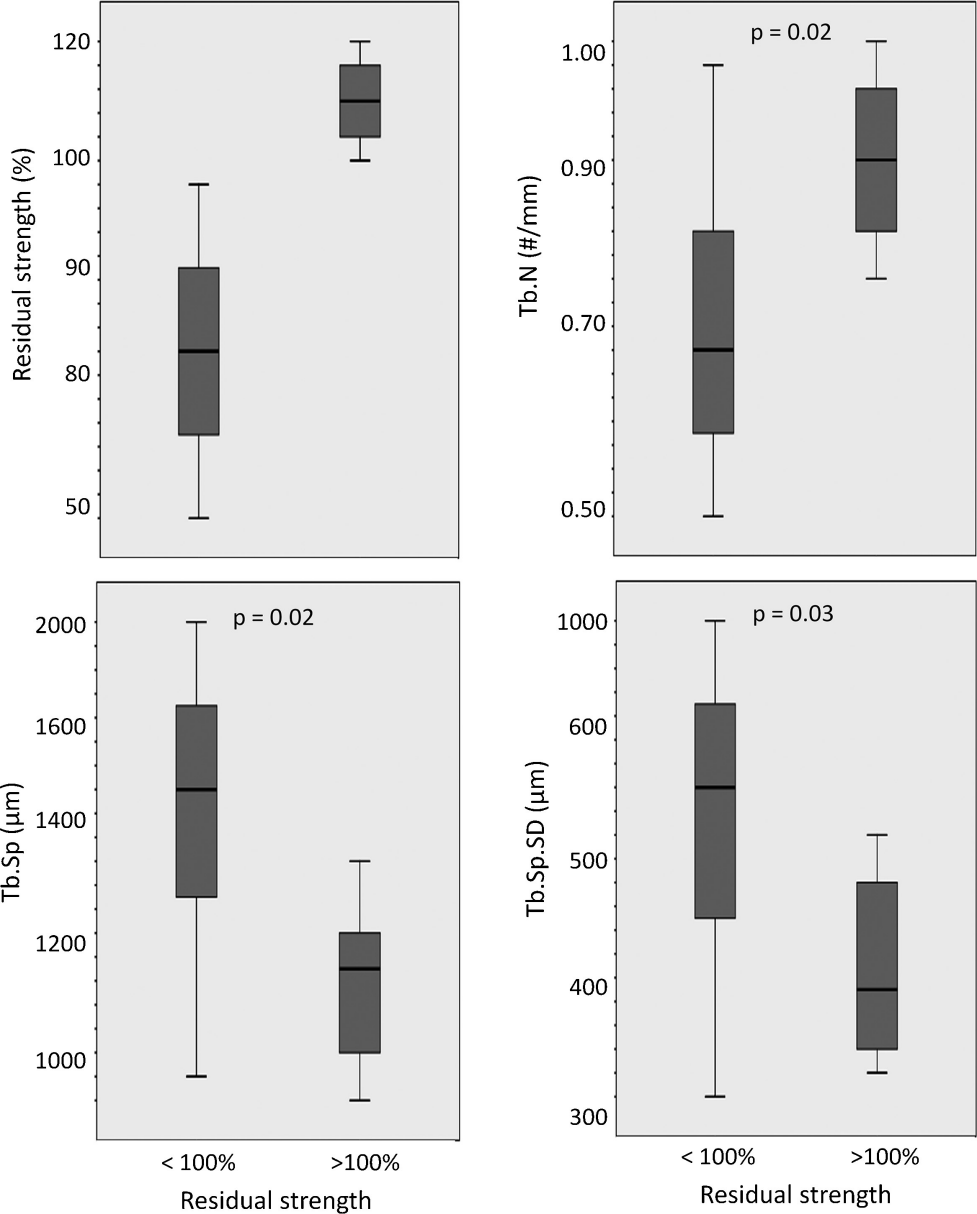
Box-plot representations of differences between vertebrae with residual strength <100% (decrease in postfracture failure load) and >100% (increase in postfracture failure load) (minimum value, lower quartile, median, upper quartile, and maximum value). The comparisons between the two groups were performed using Mann-Whitney tests.

For 6 vertebrae, postfracture failure load increased rather than decreased [residual strength = 111% ± 8% (range 101% to 121%) versus 81% ± 15% (range: 47% to 99%) for the 15 other ones]. These 6 vertebrae did not differ from the 15 other ones in term of age, sex, vertebral body height, and bone mass. However, in these 6 vertebrae, Tb.N* was significantly higher (*p* = .02) and Tb.Sp* and Tb.Sp*SD were significantly lower than the 15 other vertebrae (*p* = .02 and *p* = .03, respectively; Fig. 4).

Residual stiffness averaged 47% ± 18% (range 24% to 98%) of initial values. Residual stiffness was not correlated with bone mass (ie, BMD, BMC, and BV/TV) or with initial mechanical behavior. In addition, residual stiffness was significantly and positively correlated with Tb.Th* (*r* = 0.58, *p* = .006) and significantly and negatively correlated with Ct.Curv (|r| = −0.52, *p* = .015; Fig. 3).

## Discussion

To our knowledge, no study to date has directly assessed the ability of a whole vertebral body to carry load after a simulated mild fracture (ie, SQ grade 1). Moreover, the determinants of this postfracture load-bearing capacity are critical but poorly understood.(9,10)

We found that after sustaining an initial fracture, vertebral failure load and stiffness were decreased, whereas work to failure was increased. Although postfracture mechanical parameters were highly correlated with their initial counterparts, 47% to 71% of the variation in the postfracture mechanical behavior was explained by determinants other than the initial mechanical parameters, namely, microarchitecture.

The vertebral body consists of a trabecular bone center surrounded by a thin and porous cortical shell or perhaps trabecular condensation.(15,19,20) This complex structure can be idealized and modeled as a cellular solid such as a natural honeycomb-like material close to an open-cell plastic foam.(21) Indeed, the compressive load-displacement curve of trabecular bone is typical of this model.(21–23) The mechanical behavior shows a linear-elastic regime followed by a plateau of roughly constant load leading into a final regime of steeply rising stress. Each regime is associated with a mechanism of deformation.(22) On the first loading, the cell walls bend, giving linear elasticity, but when a critical stress is reached, the cells begin to collapse. This elastic deformation regime ends when the cell begin to collapse, giving the plastic deformation regime. Progressive compressive collapse gives a characteristic horizontal plateau of the load-displacement curve that continues until opposing cell walls meet and touch, causing the stress to rise steeply. At high loads, the cells collapse sufficiently so that the opposing cell walls touch together, and further deformation compresses the cell wall material itself. This gives the final, steeply rising portion of the load-displacement curve, labeled *densification*. An increase of relative density of the honeycomb-like structure increases the relative thickness of the cell walls. Then the resistance to cell wall bending and cell collapse goes up, giving a higher stiffness and plateau load and reducing the displacement at which densification begins.(21) This regime of densification mainly explains the increase in work to failure observed in our study between initial and postfracture mechanical behavior because cell collapse involves higher energy absorption and dissipation.

In this study, the postfracture elastic behavior was assessed by vertebral deformation recovery (VDR). VDR represents the ability of a vertebra to recover its initial height and shape—like a spring—after an initial deformation. This elastic behavior was correlated with initial work to failure, which reflects the ability of a vertebra to absorb and dissipate energy,(15) and was associated with parameters of thickness (ie, Tb.Th* and Ct.Th) but not by bone mass (ie, BMC, BMD, and BV/TV). Thus the capacity of a vertebra to recover its initial height after a simulated mild fracture depended on the capacity to dissipate energy during loading to failure, and this was mediated by the thicknesses of both the cortical shell and the trabeculae.(9) VDR also was correlated with cortical porosity (Ct.Po). However, the high correlation between Ct.Po and Ct.Th suggests that a thick “cortex” includes more endosteal bone and/or cortical remnants, leading to higher porosity—but not necessarily higher intracortical porosity. The postfracture plastic behavior was assessed by residual strength and stiffness, defined as the residual load-bearing capacity of vertebra after sustaining a fracture. The postfracture decline in stiffness was larger than the decline in failure load likely owing to the eventual impaction of failed trabeculae on themselves, thereby maintaining the postfracture failure load at a relatively higher value than the stiffness. Interestingly, the residual load-bearing capacity was independent of bone mass, as well as of initial mechanical behavior, but was explained by both cortical and trabecular microarchitecture. These results indicate that the preservation in load-bearing capacity depends on the trabecular microarchitecture more than on bone mass and initial load-bearing capacity. However, it is important to note that in our elderly population, characterized by low bone mass, the role of bone mass parameters in residual mechanical properties may be underestimated. For 6 vertebrae, postfracture failure load increased rather than decreased. These 6 vertebrae did not differ from the other vertebrae in term of age, sex, vertebral body height, and bone mass. However, in these 6 cases, trabecular microarchitecture, expressed by Tb.N*, Tb.Sp*, and trabecular microarchitecture heterogeneity (ie, Tb.Sp*SD), was significantly more robust, giving again a preponderant role to trabecular microarchitecture and its heterogeneity in preserving postfracture mechanical properties. These results suggest that drugs that preserve or enhance trabecular microarchitecture can play an important role in maintaining the mechanical properties of bone and therefore may prevent the first fracture as well as recurrence and possibly progression.(24–26) Moreover, only a small fraction of the antifracture effect of bone-resorption inhibitors can be explained by BMD gains, and thus assessment of trabecular microarchitecture may have a role not only in prediction of fracture risk but also in monitoring efficacy of antiresorptive and anabolic therapies.(24–26) The potentially positive effects of bone-resorption inhibitors on microarchitecture and subsequently on mechanical properties assumes no adverse effects of these agents on bone material properties owing to prolonged suppression of bone resorption.(26,27)

Our study had several major limitations. First, microarchitecture parameters were assessed using two high-resolution computed tomography imaging systems with different resolutions (82-µm voxel size HR-pQCT and 35-µm voxel size µCT) because our Skyscan image analyzing software did not permit the assessment of several microarchitectural parameters such as Tb.N*, Tb.Sp*, and Tb.Sp*SD. Because of partial-volume effects, lower-resolution images may lead to poor estimates of some microarchitectural features when compared with “gold standard” µCT or histomorphometry.(28–32) Although there is clear evidence for dependency of microarchitectural parameters on the scan resolution, Tb.N*, Tb.Sp*, and Tb.Sp*SD seem to be less dependent on resolution than parameters of thickness.(28,29) Indeed, a resolution reduction by a factor of 2—such as in our study between our two imaging devices—resulted in a decrease in Tb.N* of no more than 5%.(28) Moreover, several studies have compared microarchitecture measurements made with 82-µm voxel size and greater with those obtained with µCT and found very highly significant correlations between the microarchitectural parameters.(31,32) Second, the loading mode used was uniaxial compression. Because many osteoporotic vertebral fractures are anterior wedge fractures, the response to combined compression and anteroposterior (AP) bending also may be of interest.(33) Also, we did not assess the distribution of load between cortical and trabecular bone in our loading conditions in comparison with the loading conditions seen in vivo. This would be important information and highlights the necessity of further experimental and analytical studies that use finite-element analysis (FEA), both of which could extend the current experimental observations. Another limitation is that our study did not take in account other factors such as bone tissue composition (ie, degree of mineralization, collagen maturity and cross-link characteristics, and crystal size and perfection) that also contribute to vertebral mechanical properties.(33–36) Finally, our sample included vertebrae from older donors, and it is therefore not known whether these findings would apply in specimens from younger individuals with higher bone mass.

In conclusion, we found marked variation in the postfracture load-bearing capacity following simulated mild vertebral fracture. Both cortical and trabecular microarchitecture, but not bone mass, was associated with preservation of load-bearing capacity and recovery of vertebral height after an initial deformation. These results provide guidance for identifying those at highest risk for progression of vertebral fracture and suggest that therapies that prevent bone loss should preserve and enhance bone microarchitecture in order to prevent worsening of prevalent fractures and possibly delay the vertebral fracture cascade.
